# Splenomegaly versus pathological lung volume during COVID-19 infection with or without cytokine storm; a linear regression analysis using CT volumetry

**DOI:** 10.1186/s43055-022-00793-1

**Published:** 2022-05-19

**Authors:** Ahmed Samir, Heba Said Gharraf, Ayman Ibrahim Baess, Rania Ahmed Sweed, Khaled Matrawy, Mats Geijer, Adel Shalabi, Yasmine Tarek

**Affiliations:** 1 Department of Radiology, Faculty of Medicine, Alexandria University, Alexandria, Egypt; 2 Department of Chest Diseases, Faculty of Medicine, Alexandria University, Alexandria, Egypt; 3 Department of Radiology, Medical Research Institute, Alexandria University, Alexandria, Egypt; 4 Department of Radiology, Institute of Clinical Sciences, Sahlgrenska Academy, University of Gothenburg, Gothenburg, Sweden; 5 Department of Radiology, Institution of Surgical Sciences, Akademiska Hospital, Uppsala University, Uppsala, Sweden

**Keywords:** COVID, Splenomegaly, Cytokine

## Abstract

**Background:**

Due to the paucity of scientific evidence, it is unclear among pulmonologists and physicians in critical care units if and when splenomegaly in novel coronavirus disease (2019) (COVID-19) patients is worrisome. This study aims to evaluate the significance of splenic volume during COVID-19 infection with or without cytokine storm and correlates splenic volume to the volume of pathological lung changes through linear regression analysis.

**Results:**

A retrospective study collected 509 polymerase chain reaction proved COVID-19 patients (399 males, 110 females; mean age 48 years, age range 24–78 years) between June and November 2021, without a history of splenic pathology. A control group of age and sex-matched 509 healthy subjects was used and analyzed according to the splenic volume. Five consulting radiologists evaluated initial and follow-up computed tomography (CT) examinations using lung CT volumetry and splenic volume calculation in consensus. Three consulting pulmonologists correlated the severity of clinical and laboratory findings, including oxygen requirements and interleukin-6 (IL-6) levels. The *T* test results for comparison between the COVID-19 patients and the healthy subjects control group regarding the splenic volume were significant (*T* value was − 4.731452 and *p* value was 0.00002). There was no significant correlation between the severity of the disease and normal-sized spleen (26% of patients, *p* = 0.916) or splenomegaly (24% of patients, *p* = 0.579). On the other hand, all patients with a small spleen or progressive splenomegaly during serial follow-up imaging had clinically severe disease with a statistically significant correlation (*p* = 0.017 and 0.003, respectively). Ninety-seven percent of patients with clinically mild disease and splenomegaly had 0–20% lung involvement (CT-severity score 1) while all patients with clinically severe disease and splenomegaly had 27–73% lung involvement (CT-severity score 2 and 3) (*r* = 0.305, *p* = 0.030).

**Conclusions:**

Splenomegaly is a non-specific sign that may be found during mild and severe COVID-19 infection, it was not statistically correlated with the clinical severity and a weak positive relationship was found between the splenic size and the CT-severity score of the pathological lung volume. On the other hand, the presence of splenic atrophy or progressive splenomegaly was correlated with severe COVID-19 presentation and “cytokine storm”. Therefore, the splenic volume changes should not be overlooked in COVID-19 serial CT examinations, particularly in severe or critically ill patients with cytokine storms.

## Background

The spleen is a secondary lymphoid organ responsible for hematopoiesis and immune surveillance, in addition to the process of maturation and storage of both B-cell and T-cell lymphocytes with the consequent manufacturing of immunoglobulin-G (IgG) antibodies. Splenomegaly means splenic enlargement, usually when exceeding a volume of 315 cc or a weight of 400 g. Massive splenomegaly usually means splenic enlargement exceeding 1000 g [1–3].

The causes of splenomegaly are numerous and one of them is viral infection, including COVID-19, because of lymphoid hypertrophy. This could be attributed to either a direct viral attack with replication resulting in T-cell damage and lymphopenia or thrombocyte sequestration with thrombocytopenia [4].

Splenomegaly is one of the parameters described for the diagnosis of the “cytokine storm” that can complicate many diseases, including COVID-19 infection. This “cytokine storm” means excess production of pro-inflammatory cytokines and results in multi-organ damage with rapid deterioration of the clinical situation and poor prognosis [5–7]. It has been suggested as a cause for rapid patient deterioration in COVID-19 infection [8, 9].

Due to the paucity of scientific evidence, it is currently unclear among pulmonologists and physicians in critical care units if and when splenomegaly in COVID-19 patients is worrisome. Few pieces of literature discussed the role of CT in measuring splenic volume changes during COVID-19 infection and correlating this volume to the clinical severity and pathological lung volume [10].

The current study aimed to evaluate the significance of splenic volume changes during COVID-19 infection with or without cytokine storm and to correlate the CT-calculated splenic volume to clinical severity and CT-severity score through linear regression analysis.

## Methods

### Study population and ethical protocol

The study was approved by The Ethics Committee at the authors’ institution. The need for informed patient consent was waived by the Research Ethics Board on the assurance of the confidentiality of patient information and medical records.

- Inclusion criteria were patients with (1) a positive PCR test for COVID-19 infection, (2) available clinical and laboratory data including the oxygen saturation and oxygen therapeutic requirements in addition to the D-dimer and interleukin-6 (IL-6) levels, and (3) available initial and follow-up CT examinations for the chest and upper abdomen.
- Exclusion criteria were (1) primary splenic diseases such as infection, infarction, or mass, (2) secondary splenic involvement in immunologic or hematologic or hepatic diseases, and (3) poor quality of CT images.

In a multi-center retrospective analysis of patients treated for COVID-19 infection between June and November 2021, 568 consecutive patients with positive PCR results for COVID-19 were initially considered for inclusion.

After the exclusion of 59 patients for liver cirrhosis, autoimmune, and blood diseases that can influence the splenic size, the patient cohort comprised 509 patients (399 males and 110 females, mean age 48 years ± 13 standard deviation (SD), age range 24–78 years). History of smoking was positive in 145/509 patients. Hypertension, renal and neoplastic diseases were reported in 87/509, 43/509, and 22/509 patients respectively.

A control group of age and sex-matched 509 healthy subjects was used and analyzed according to the splenic volume.

### Classification of patients according to the clinical severity

Three expert consulting pulmonologists (experience 19–21 years, respectively) were responsible for the classification of the included patients according to clinical severity.

They evaluated certain clinical and laboratory parameters including patient oxygen saturation and oxygen therapeutic requirements in addition to the D-dimer and interleukin-6 (IL-6) levels.

They used the World Health Organization (WHO) criteria for severe or critical clinical COVID-19 infection as follows: (1) Oxygen (O_2_) saturation in room air < 93%, (2) external O_2_ support ranging from high flow nasal O_2_ up to mechanical ventilation, and (3) high D-dimer levels (> 500 ng/ml). The initiation of “cytokine storm” parameters further included (1) sudden oxygen desaturation, (2) poorly controlled or relapsing high fever, and (3) elevated IL-6 levels (> 80 pg/ml) [11, 12].

### Assessment of pulmonary and splenic changes in CT

Two types of CT scanners were used, one SOMATOM Sensation 64 (Siemens, Erlangen, Germany) and one Aquilion CXL/CX 128 (Toshiba, Canon Medical Systems, Tustin, CA, USA). The CT scanning parameters were: slice thickness; 1–1.25 mm, tube rotation; 0.6–0.9 s, detector collimation; 1 mm, FOV; 350 mm × 350 mm, tube voltage; 120–130 kVp, and tube current; 200 mA. Intravenous contrast was not used.

The pulmonary and splenic changes in CT images were assessed in consensus by five consulting radiologists (14–26 years of experience with chest and abdominal CT) at the same time as follows:

- The pulmonary assessment was combined morphologic and volumetric:
  - A pulmonary multi-planar reformation (MPR) review was performed using OsiriX MD 11.0 software (Pixmeo SARL, Geneva, Switzerland). Universal CT characteristics of COVID-19 were traced on the images, including ground-glass opacities with or without consolidation, the reverse halo or “Atoll” sign [10] which represents peripheral organization surrounding a ground-glass patch, and the “Crazy-paving pattern” [13] which represents mixed ground-glass opacities and super-added inter-lobular septal thickening.
  - Lung volumetric assessment was performed using also OsiriX MD 11.0 software with ROI 2D/3D reconstruction and threshold interval adjustment or a Vitrea workstation (Toshiba, Canon Medical Systems, Tustin, CA, USA) with computed volume calculation. A universal CT severity scoring was used where Score 1 referred to 0-25% lung involvement, Score 2 to 26-50%, Score 3 to 51–75%, and Score 4 to > 75% lung involvement [14].
- The splenic assessment was combined two and three dimensional:
  - The splenic diameters were measured as AP and side-to-side diameters on axial sections and the craniocaudal diameter on coronal sections.
  - The splenic volume was automatically calculated by a Vitrea workstation (Toshiba, Canon Medical Systems, Tustin, CA, USA).
  - Because of the variable ranges for splenic two-dimensional (2D) measurements, the splenic volume was used for the accurate determination of splenomegaly.
  - Normal splenic volume ranges from 107 to 315 cc. Splenic enlargement refers to a volume >315 cc. Splenic massive enlargement refers to a volume > 1000c. Splenic volume may vary according to patient age, weight, and body mass index, but not in a significant way [1–3].

### Statistical analysis

- Mean, mode, median, variance, and standard deviations were calculated.
- Statistical significance was analyzed by Pearson correlation coefficient (*r*) and linear regression analyzes.
- Chi-square analysis was performed and a *p* value < 0.05 was taken to show statistical significance between examined groups of patients according to their age, clinical severity, size of the spleen, and volume of pathological lungs.
- *T* test (at *p* < 0.05), regarding the splenic volume, was utilized for comparison between the group of infected patients and the control group of healthy subjects.
- Two online calculators were used: Alcula online calculators (http://www.alcula.com/calculators/statistics/) and (https://www.socscistatistics.com).

## Results

Most patients were in the fifth decade of life. The detailed distribution of patients according to age is shown in (Table 1)*.*

**Table 1:** Distribution of patients according to their age

Age	Number of patients
21–30	30 (6%)
31–40	128 (25%)
41–50	153 (30%)
51–60	116 (23%)
61–70	30 (6%)
71–80	52 (10%)
Total	509

### Comparison between the COVID-19 patients' group and the healthy subjects control group regarding the splenic volume

Normal-sized spleen (ranging from 107 to 315 cc) was found in 250/509 (49%) COVID-19 patients and 481/509 (94.5%) healthy subjects control group*.*

Mild splenomegaly (ranging from 315 to 1000 cc) was found in 245/509 (48%) COVID-19 patients and 481/509 (94.5%) healthy subjects control group.

Massive splenomegaly (> 1000 cc) was found in 5/509 (1%) COVID-19 patients and not found in the healthy subjects control group.

Small-sized spleen (< 107 cc) was found in 9/509 (2%) COVID-19 patients and not found in the healthy subjects control group.

### Splenic and lung volumetry in correlation to clinical severity (Table 2).

**Table 2:** Clinical parameters in patients with small, normal-sized and enlarged spleens, respectively, with clinically mild or severe Covid-19 infection

Clinical parameter	Normal-sized spleen, clinically mild disease	Normal-sized spleen, clinically severe disease	Small spleen, clinically severe disease	Splenomegaly, clinically mild disease	Splenomegaly, clinically severe disease
Splenic size	*n* = 250 (49%)		*n* = 9 (2%)	*n* = 250 (49%)	
Clinically mild disease, O2 Sat ≥ 93%	*n* = 192 (76%)			*n* = 184 (74%)	
Clinically severe disease, O2 Sat < 93%		*n* = 58 (24%)	*n* = 9 (100%)		*n* = 66 (26%)
Pathological lung volume	97% of patients → 0–20% (score 1)	All patients → 23–71% (score 1–3)	All patients → 34–55% (score 2 & 3)	97% of patients → 0–20% (score 1)	All patients → 27–73% (Score 2 & 3)
Splenic volume at follow-up CT	100% unchanged	100% unchanged	100% unchanged	87% unchanged, 13% regression	75% unchanged, 25% progression

#### Generally

The clinically severe patients with normal-sized spleen had pathological lung volume > 23%, meanwhile, the clinically severe patients with splenomegaly had pathological lung volume > 27%.

Mild splenomegaly was noted in clinically mild and severe patients. Massive splenomegaly (> 1000 cc) was noted only in severe patients.

##### Patients with a normal-sized spleen

**Fig. 1:**
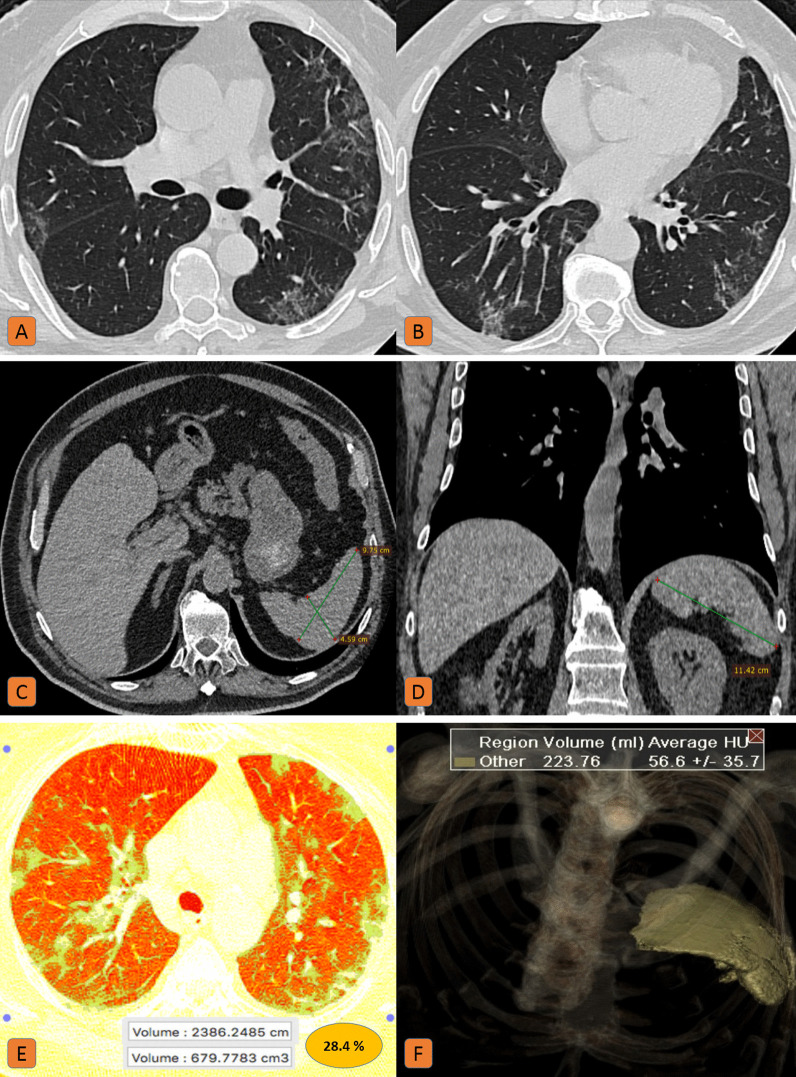
Normal sized spleen in a 74-year-old male patient with severe COVID-19 infection: **A**, **B** Axial chest CT (lung window) showed bilateral sub-pleural ground-glass patches with mild reticular thickening. **C** Axial, and **D** coronal CT cuts of the upper abdomen (soft tissue window) showed normal splenic diameters. **E** Lung volumetric CT assessment revealed 28.5% pathological lung involvement. **F** CT volumetry of the spleen was 223 cc

Two hundred and fifty patients (49%) had a spleen of normal size. Around 24% of these patients had clinically severe disease with high IL-6 levels (around 150–300 ng/ml) denoting cytokine storm and quantitative lung involvement ranging from 23 to 71% (CT-severity score 1–3) (Fig. 1). At follow-up CT, the splenic size remained unchanged.

##### Patients with a small-sized spleen

Nine patients (2%) had a small spleen (longest diameter < 7 cm and volume < 150 ml). All of these patients had clinically severe disease with high IL-6 levels (ranging between 360 and 520 ng/ml) denoting cytokine storm. Their quantitative lung involvement ranged between 34 and 55%. At follow-up CT, the splenic size remained unchanged.

##### Patients with splenomegaly

**Fig. 2:**
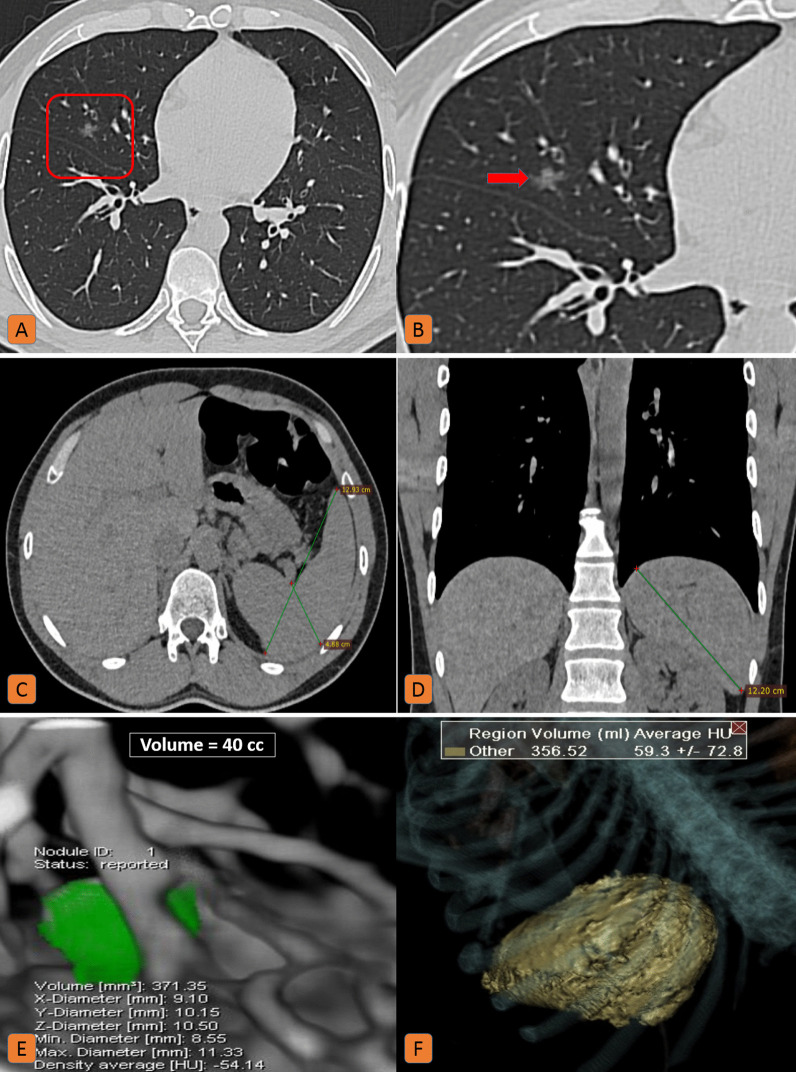
Splenomegaly in a 35-year-old male patient with mild COVID-19 infection. **A** Axial chest CT (lung window) and **B** zoomed image showed single right middle small ground-glass nodular opacity. **C** Axial, and **D** coronal CT cuts of the upper window (soft tissue window) showed splenic 2D diameters (13 × 4.6 × 12.4 cm) (anteroposterior diameter x side to side thickness x craniocaudal diameter). **E** Lung volumetric CT assessment revealed a 40 cc lung nodule. **F** CT volumetry of the spleen was 356 cc

**Fig. 3:**
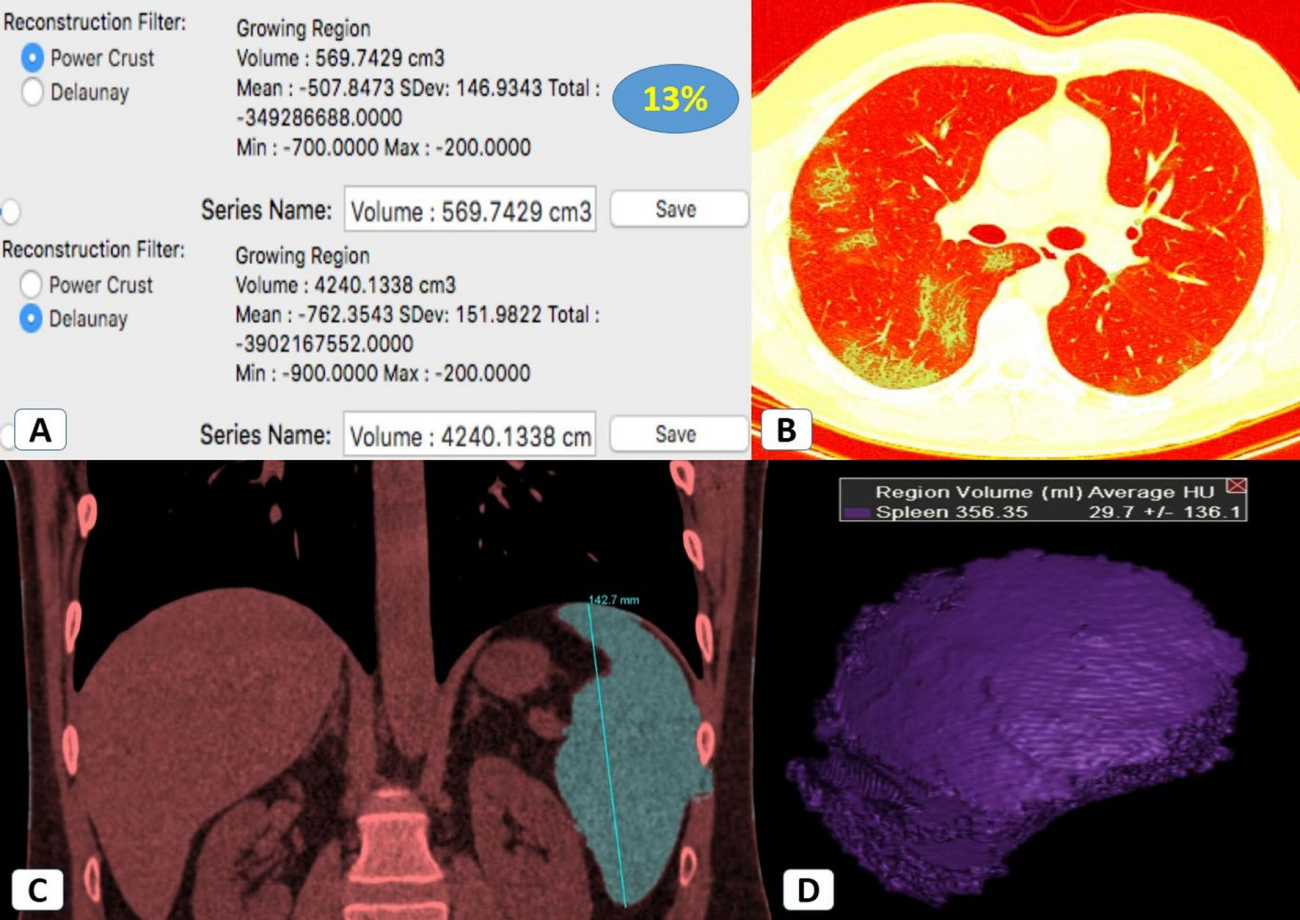
Splenomegaly in a 33-year-old male patient with mild COVID-19 infection: **A**, **B** Volumetric CT assessment of the lung revealed 13% pathological lung involvement. **C** Coronal CT (mediastinal window) showed an increased craniocaudal bi-polar diameter of the spleen (14.2 cm). **D** CT volumetry of the spleen was 356 cc

**Fig. 4:**
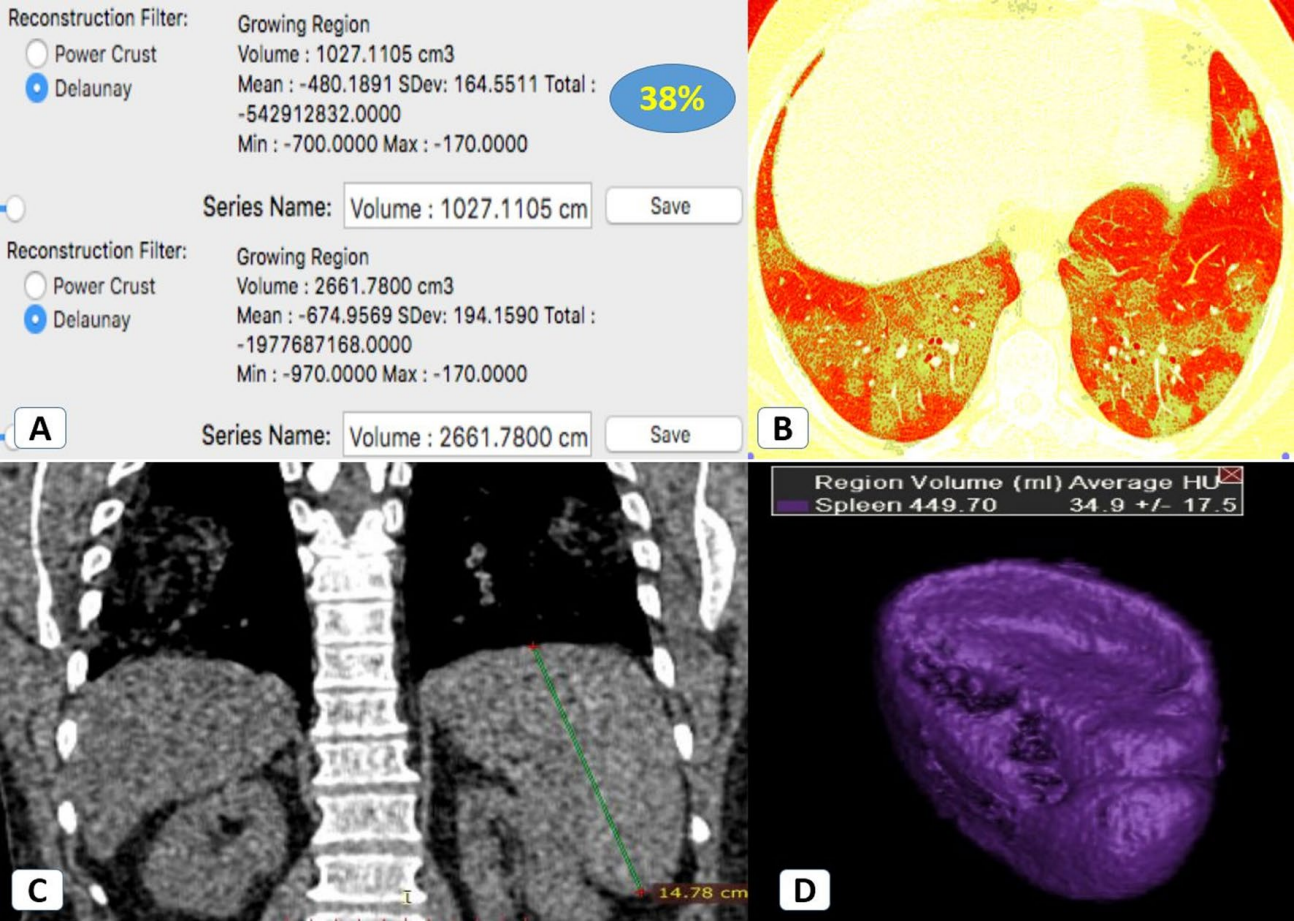
Splenomegaly in a 58-year-old female patient with severe COVID-19 infection: **A**, **B** Volumetric CT assessment of the lung revealed 38% pathological lung involvement. **C** Coronal CT mediastinal window showed an increased craniocaudal bi-polar diameter of the spleen (14.8 cm). **D** CT volumetry of the spleen was 450 cc

The remaining 250 patients (49%) had splenomegaly (splenic volume ranged from 300 to 1020 cc). Around 74% of these patients were clinically mild (Fig. 2 and 3), meanwhile the remaining 26% had a clinically severe disease with high IL-6 levels (around 150–700 ng/ml) denoting cytokine storm and a quantitative lung involvement ranging from 27 to 73% (CT-severity score 1–3) (Fig. 4).

Additionally, regarding the 2D measurements of the enlarged spleens, the splenic craniocaudal (CC) length ranged from 11.5 to 16 cm, the anteroposterior (AP) splenic width from 11.2 to 14 cm, and the splenic side-to-side (SS) thickness from 4.2 cm to 5.9 cm.

Based on mean, variance, and standard deviation data analysis, the degree of splenomegaly was slightly higher in patients with clinically severe disease (1.1:1) without reaching statistical significance.

At follow-up CT of patients with clinically mild disease and splenomegaly, the splenic size remained almost unchanged in 160/184 patients (87%) and decreased in 24/184 patients (13%).

**Fig. 5:**
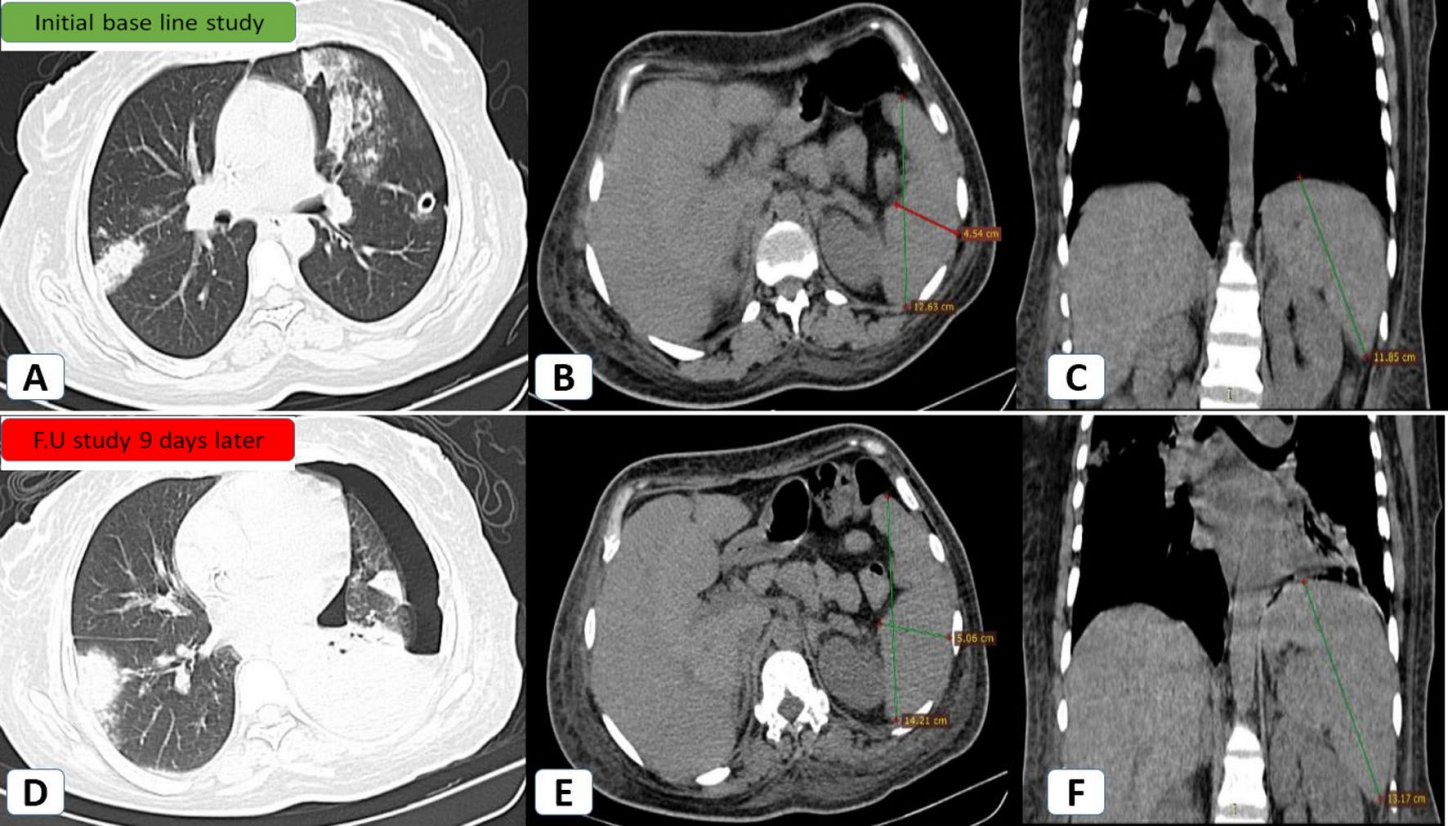
Progressive splenomegaly in a 43-year-old female patient with severe COVID-19 infection and elevated Interleukin-6 (320 pg/ml), consistent with initiation of cytokine storm: **A**–**C** Initial baseline study: **A** Axial HRCT (lung window) showing right posterior segmental upper lobar sub-pleural ground glass patch and left upper lobar confluent ground opacities. Minimal left-sided pneumothorax was noted with an inserted inter-costal tube. **B** Axial CT (mediastinal window) for the maximum anteroposterior and side-to-side splenic dimensions (12.6 cm and 4.5 cm). **C** Coronal CT (mediastinal window) for the maximum splenic craniocaudal dimension (11.8 cm). **D**–**F** Follow-up of the same patient nine days later: **D** Axial HRCT (lung window) showing left-sided progressing hydro-pneumothorax with denovo left lingular sub-pleural wedge-shaped patch. **E** Axial CT (mediastinal window) for the maximum anteroposterior and side-to-side dimensions (14.2 cm and 5 cm). **F** Coronal CT (mediastinal window) for the maximum craniocaudal dimension (13.1 cm)

At follow-up CT of patients with clinically severe disease and splenomegaly, splenic size remained almost unchanged in 49/66 patients (75%) and increased in 17/66 patients (25%) (Fig. 5).

The detailed distribution of mild and severe/critically ill patients with splenomegaly according to splenic measurements and lung volumetry is shown in (Table 3).

**Table 3:** Distribution of patients with splenomegaly according to splenic volume (cc) and lung CT volumetry among mild and severe patients

	Mild (184/250 patients)	Severe-critical (66/250 patients)
According to splenic volume (cc)		
300-	76/41.3%	7/10.6%
400-	61/33.2%	18/27.3%
500-	13/7.1%	22/33.3%
600-	23/12.5%	14/21.2%
700-	11/6%	0
800-	0	0
900-	0	0
1000-	0	5/7.6%
Mean	479.316	549
Median	442	516.5
Mode	348	390
SD	126.357	160.154
According to lung CT volumetry		
0–10%	131/71.2%	0
11–20%	48/26.1%	0
21–30%	5/2.7%	18/27.3%
31–40%	0	33/50%
41–50%	0	0
51–60%	0	5/7.6%
61–70%	0	5/7.6%
71–80%	0	5/7.6%

#### *T* test and linear regression results

*T* value was − 4.731452 and *p* value was 0.00002. Therefore the results of the *T* test for comparison between the group of infected patients and the control group of healthy subjects regarding the splenic volume were considered significant.

No significant relationship was shown between splenic size and clinical severity for patients with normal splenic size (*p* = 0.916) or splenomegaly (*p* = 0.579). On the other hand, there was a significant statistical relationship between splenic size and clinical severity for patients with a small spleen (*p* = 0.017) and patients with progressive splenomegaly (*p* = 0.003).

**Fig. 6:**
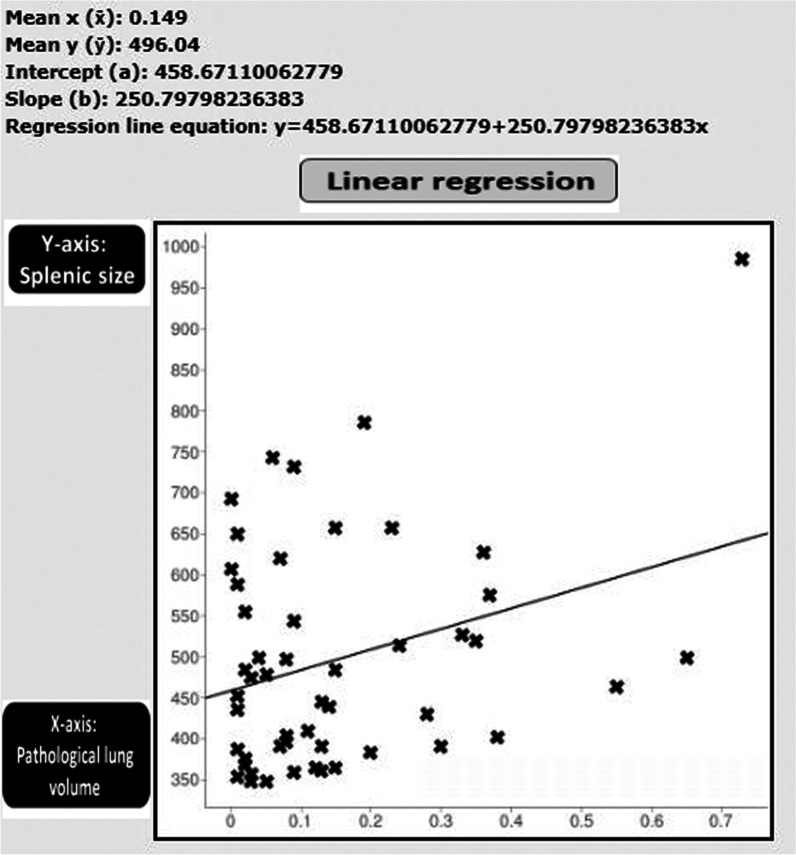
Schematic presentation of Pearson correlation coefficient and linear regression analysis between the amount of pathological lung volume (*X*-axis) and splenic size (*Y*-axis): A positive (but weak) relationship was shown (*r* = 0.3047, *p* = 0.02989)

A positive, but weak significant relationship was shown between the amount of pathological lung volume and splenic size (*r* = 0.3047, *p* = 0.02989) (Fig. 6).

**Fig. 7:**
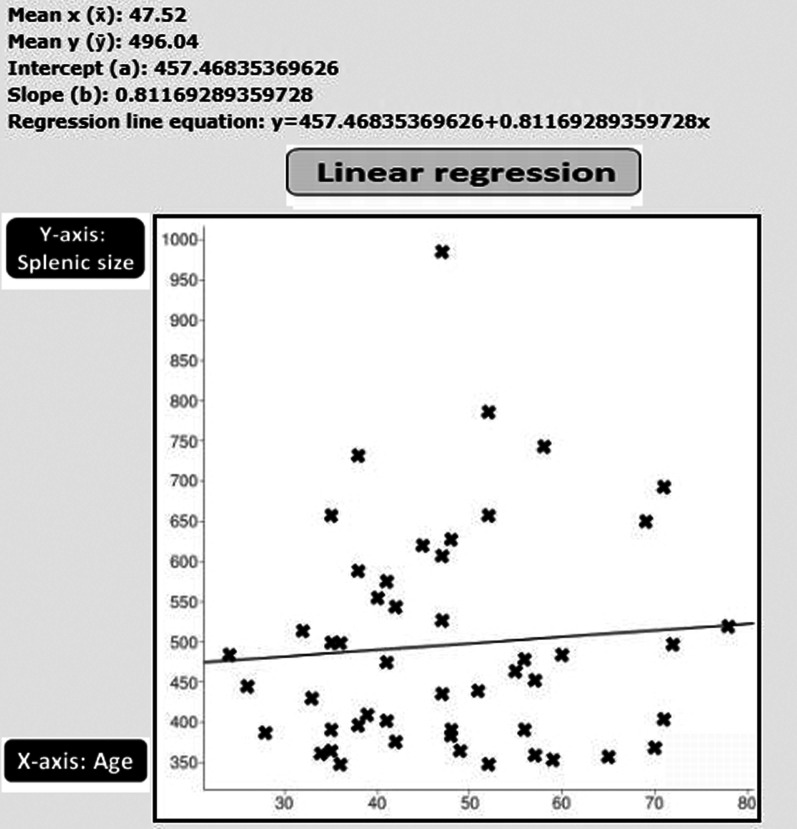
Schematic presentation of Pearson correlation coefficient and linear regression analysis between the patients’ age (*X*-axis) and splenic size (*Y*-axis): no statistical relationship was shown (*r* = 0.0771, *p* = 0.594599)

**Fig. 8:**
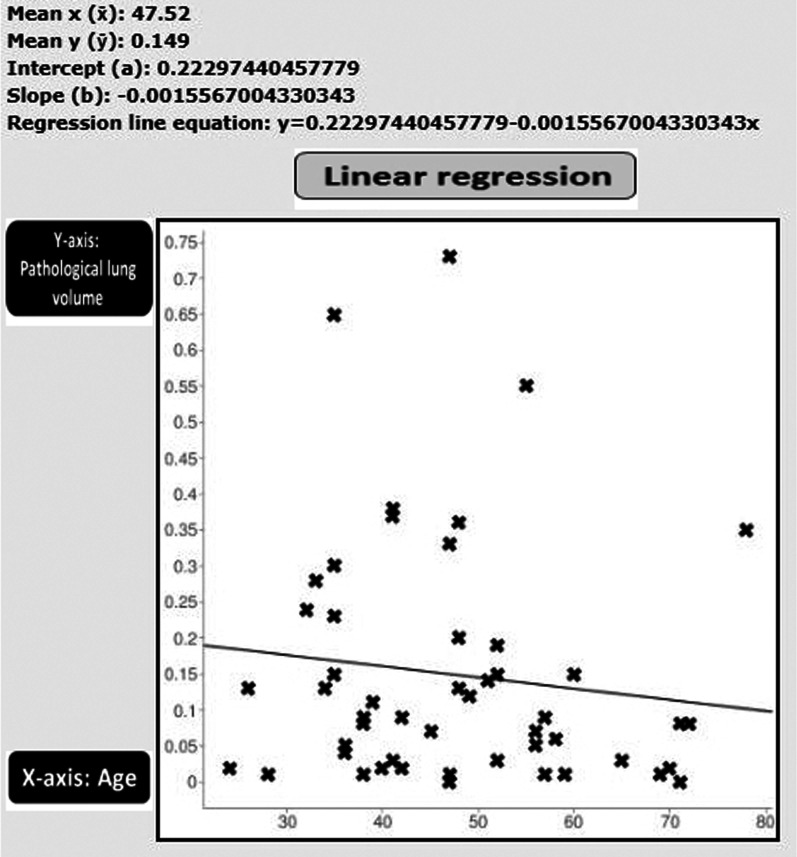
Schematic presentation of Pearson correlation coefficient and linear regression analysis between the patients' age (*X*-axis) and the number of pathological lung changes (*Y*-axis): No statistical relationship was shown (*r* = − 0.1206, *p* = 0.406498)

No statistical relationship was shown between the patients’ age and the splenic size (*r* = 0.0771, *p* = 0.594599) (Fig. 7). Nor was any statistical relationship shown between the patients’ age and the volume of pathological lung changes (*r* = − 0.1206, *p* = 0.406498) (Fig. 8).

**Fig. 9:**
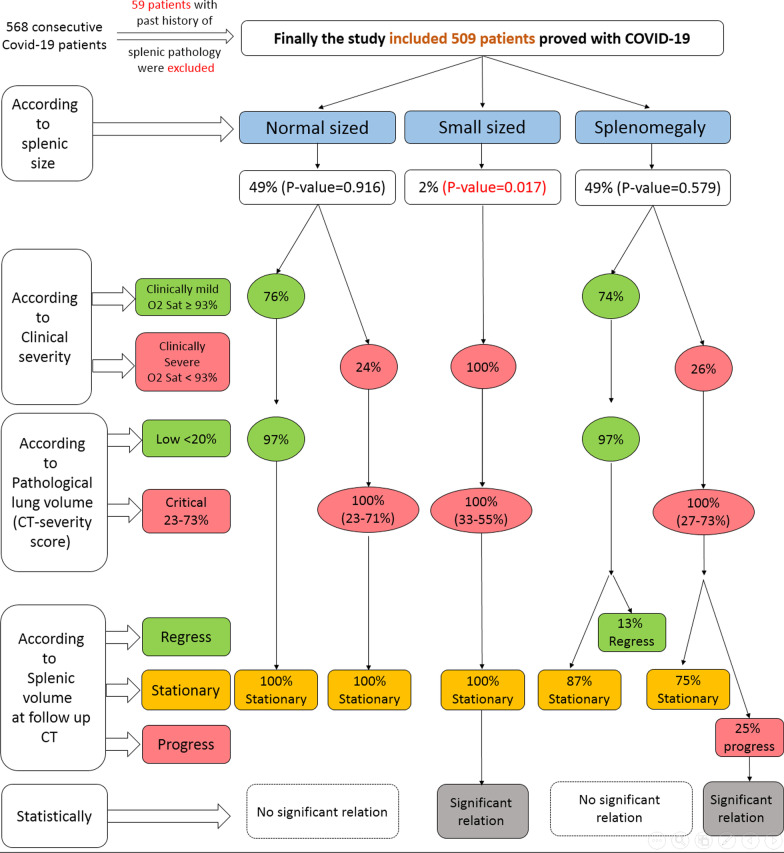
A chart summarizing the results of the study regarding splenic size, clinical severity, pathological lung volume, follow-up, and statistical results

A full detailed chart is summarizing the results of the study regarding splenic size, clinical severity, pathological lung volume, follow-up, and statistical results (Fig. 9).

## Discussion

Splenomegaly in COVID-19 patients can occur either early because of a direct viral attack on the spleen or late due to a generalized attack of the body’s immune system on its tissues [15].

Only a few reports in the literature have discussed the incidence of splenomegaly in COVID-19 patients. Furthermore, the significance of splenomegaly during COVID-19 infection, especially during cytokine storms, has been also unclear.

In the current study, the authors estimated the incidence of splenomegaly in COVID-19 patients. Additionally, they evaluated the significance of splenic volume changes during mild and severe COVID-19 infection with or without cytokine storm. They also correlated the CT-calculated splenic volume with the CT-severity score of pathological lung parenchyma through linear regression analysis.

### The incidence of splenomegaly in COVID-19

The incidence of splenomegaly in COVID-19 patients in this study (49%) approximated that reported in the study by Shiralkar et al. [16] (58%) but exceeded that reported as 18% by Tabatabaei et al. [17] and as being uncommon by Xu et al. [18]. The incidence of splenomegaly in the critically ill COVID-19 patients in this study (49%) also exceeded that reported by Tabatabaei et al. [17] (18%).

This difference could be explained by the fact that differences may result from different studies applying different measuring techniques. In this study, 3D volumetric measurements replaced the routine linear 2D splenic measurements in the analysis of the splenic volume. This explanation typically matches Lamb et al. [19].

Similar to that reported by Shiralkar et al. [16], Tabatabaei et al. [17], and Li et al. [20], mild splenomegaly in this study predominated COVID-19 infection while massive splenomegaly was rare (2%).

### The significance of splenic volume changes in COVID-19

Splenomegaly, in this study, was not significantly correlated with the clinical severity in COVID-19 patients. Furthermore, the relationship between splenic size and the CT-severity score of the pathological lung volume was weakly positive. This typically matches the findings reported by Xu et al. [18] and Gao et al. [21].

Conversely, progressive splenomegaly or atrophic spleen, in this study, was significantly correlated with the clinical severity in COVID-19 patients. This was also depicted in the study by Xu et al. [18] and Gao et al. [21].

This is mostly explained by the hyper-immune response at the initiation and progress of the cytokine storm, resulting in progressive splenomegaly. Later in the disease, delayed immune system exhaustion took place altogether with solid organ and lymphoid damage, resulting in splenic atrophy.

### Limitations and strengths

The study may be limited by the variability regarding sex incidence (four to one male to female incidence ratio).

On the other hand, this study added to the literature when described the significance of splenic volume changes in severe forms of COVID-19 with cytokine storms. This was achieved by serial radiological assessment side-by-side with the clinical presentation and management plans with the exact splenic volume measurements and detailed pathologic lung volume measurements.

## Conclusions

Splenomegaly is a non-specific sign that may be found during mild and severe COVID-19 infection, it was not statistically correlated with the clinical severity and a weak positive relationship was found between the splenic size and the CT-severity score of the pathological lung volume.

On the other hand, the presence of splenic atrophy or progressive splenomegaly was correlated with severe COVID-19 presentation and “cytokine storm”. Therefore, the splenic volume changes should not be overlooked in COVID-19 serial CT examinations, particularly in severe or critically ill patients with cytokine storms.

## Data Availability

The datasets used and/or analyzed during the current study are available from the corresponding author on reasonable request.

## Abbreviations

COVID-19: Novel coronavirus disease (2019)
PCR: Polymerase chain reaction
CT: Computed tomography
IL-6: Interleukin-6
MPR: Multi-planar reformation
